# Takotsubo Syndrome during Pertuzumab and Trastuzumab Therapy for HER2-Positive Metastatic Breast Cancer

**DOI:** 10.3390/biomedicines12010179

**Published:** 2024-01-14

**Authors:** Azzurra Irelli, Laura Ceriello, Leonardo Valerio Patruno, Alessandra Tessitore, Edoardo Alesse, Katia Cannita, Donatello Fabiani

**Affiliations:** 1 Medical Oncology Unit, Department of Oncology, “Giuseppe Mazzini” Hospital, AUSL 04 Teramo, 64100 Teramo, Italy; leonardovalerio.patruno@aslteramo.it (L.V.P.); katia.cannita@aslteramo.it (K.C.); 2 Cardiology Unit, Department of Cardiovascular Diseases, “Giuseppe Mazzini” Hospital, AUSL 04 Teramo, 64100 Teramo, Italy; laura.ceriello@aslteramo.it (L.C.); donatello.fabiani@aslteramo.it (D.F.); 3 Department of Biotechnological and Applied Clinical Sciences, University of L’Aquila, Via Vetoio, 67100 L’Aquila, Italy; alessandra.tessitore@univaq.it (A.T.); edoardo.alesse@univaq.it (E.A.)

**Keywords:** pertuzumab, trastuzumab, fulvestrant, denosumab, cardiac toxicity, Takotsubo syndrome

## Abstract

Pertuzumab and trastuzumab have been shown to improve the outcomes of patients with metastatic breast cancer, with a rate of left ventricular dysfunction of approximately 6%. We report the case of a postmenopausal woman who presented with Takotsubo syndrome during maintenance therapy with pertuzumab and trastuzumab, in association with fulvestrant (an anti-estrogen) and denosumab. After normalization of cardiac function, therapy with pertuzumab and trastuzumab was resumed in the absence of new cardiac toxicity. We report the first clinical case of Takotsubo syndrome during double anti-HER2 blockade in association with an antiestrogen. Furthermore, we show how anti-HER2 therapy can be safely resumed after the detection of Takotsubo syndrome.

## 1. Introduction

Human epidermal growth factor receptor 2 (HER2) is a transmembrane tyrosine kinase receptor belonging to the HER receptor family and is overexpressed in approximately 15–25% of breast cancer (BC) cases. Overexpression of the HER2 protein or amplification of the *HER2* gene is associated with a poor prognosis, i.e., reduced progression-free survival and overall survival [1,2]. Preclinical and clinical studies demonstrate that dual anti-HER2 therapy with trastuzumab and pertuzumab provides a complete blockade of HER2 signaling and improves the objective response and clinical outcomes. Cardiotoxicity, characterized by left ventricular (LV) systolic dysfunction with or without symptoms and/or signs of heart failure, represents the most worrying adverse event during anti-HER2 therapy. The damage induced by anti-HER2 therapy is transient and generally reversible upon the discontinuation of treatment [3].

Numerous clinical studies evaluated the risk of cardiotoxicity of trastuzumab, both in monotherapy and in combination with pertuzumab. 

In a meta-analysis of randomized and cohort studies, the risk of severe cardiotoxicity with trastuzumab was estimated to be 3.14% in all patients with metastatic BC, but the risk was consistently higher among patients enrolled in real-life cohort studies compared to randomized ones. The percentage increased from 3.00 to 3.68% when trastuzumab was used as a first-line treatment and in additional lines of therapy, respectively. Furthermore, the risk of serious cardiotoxicity was substantially higher among patients with cardiovascular risk factors, with a prevalence of 19.1% among patients with a history of pre-existing heart disease [4].

In the Cleopatra study, dual anti-HER2-targeted therapy (pertuzumab and trastuzumab) plus docetaxel did not increase the incidence of LV systolic dysfunction compared to that in the control arm (trastuzumab plus docetaxel). The rate of LV dysfunction was 6.6% in the pertuzumab group compared to 8.6% in the control group. Reductions in left ventricular ejection fraction (LVEF) of 10% or more from the baseline to an absolute value less than 50% occurred in 6.1% of patients in the pertuzumab group and 7.4% of patients in the control group [5].

In the study by Yu et al., the combination of paclitaxel, pertuzumab, and trastuzumab caused a significant decline in the LVEF of ≥10 absolute percentage points to <50% in 3.8% of patients compared to 6.6% of patients in the paclitaxel and trastuzumab arm, and the incidence of symptomatic LV systolic dysfunction was low in both groups (1.0% versus 1.8%, respectively) [6].

Takotsubo syndrome (TS) is a reversible cardiomyopathy first reported by Sato and colleagues in 1990 [7,8]. The name of this syndrome derives from the Japanese term, which indicates an amphora with a narrow neck and a round bottom used for catching octopuses. The shape of this amphora recalls that of the LV of patients suffering from this cardiomyopathy and which can be documented with transthoracic echocardiography (TTE) or via ventriculography. The peculiar aspect is characterized by hypo/akinesia of the apical segments of the LV in the presence of normo- or hyperkinesia of the basal segments, which generally occurs following exposure to physical or emotional triggers. For these reasons, TS is also called stress cardiomyopathy, broken heart syndrome, or apical ballooning syndrome. 

In 2006, the American Heart Association incorporated this pathology into the group of acquired cardiomyopathies [9,10]. TS has been described in cancer patients, and it has been attributed to the physiological and emotional stress associated with cancer diagnosis and anticancer treatment. 

It is possible that combination therapy with anti-HER2 antibodies may increase the risk of occurrence of this rare event [11].

## 2. Materials and Methods

### 2.1. Case Presentation

We present the case of a female patient with HER2-positive metastatic BC treated with dual anti-HER2 therapy (pertuzumab and trastuzumab), fulvestrant and denosumab at the Medical Oncology Unit of “Giuseppe Mazzini” Hospital, Teramo (Italy). The patient provided written consent for publication.

### 2.2. Systematic Review

A PubMed search was conducted on 17 November 2023, using the query “takotsubo syndrome AND (trastuzumab OR pertuzumab)”. Additional articles were selected by reading the identified articles (Figure S1).

The aim of this work was to provide an overview of TS in patients with BC treated with anti-HER2 therapy and describe the experience of our center. The clinical case presented is interesting because it is the second to report TS in a patient treated with pertuzumab and trastuzumab, but the first on double anti-HER2 blockade in association with the anti-estrogen fulvestrant.

### 2.3. Diagnostic Criteria

Electrocardiogram (ECG). Both the ECG performed at the onset of symptoms and their evolution are distinctive of TS. In particular, the first ECG usually shows new-onset repolarization abnormalities, namely diffuse and deep T wave inversion and QTc prolongation. Such alterations progressively decrease on subsequent ECGs. 

Echocardiography. Patients usually show transient alterations of wall motion contractility, involving the apical segments of the LV beyond a single epicardial vessel distribution.

Cine-ventriculography. This procedure represents the gold-standard technique for TS diagnosis. This procedure typically reveals an apical and mid-ventricular hypokinesia in end-systolic frames in the presence of hypercontractility of the basal segments.

Coronary angiography. This shows the absence of coronary artery obstructive lesions, thus excluding an ischemic etiology of the clinical presentation. 

Cardiac biomarkers. A moderate increase in troponin and natriuretic peptides is common in TS and is included in the InterTak diagnostic criteria to corroborate the diagnosis.

Cardiac magnetic resonance imaging (CMRI). The pathognomonic characteristics of TS are represented by a diffuse edema, mainly in the apical dysfunctional segments evidenced on T2-weighted sequences on MRI scans performed in the acute phase. The transient and reversible nature of this cardiomyopathy is confirmed by the resolution of edema on follow-up scans and, particularly, by the absence of late gadolinium enhancement with a non-ischemic distribution on post-contrast images [12]. 

## 3. Results

### 3.1. Case Presentation

In May 2022, a 56-year-old woman in clinical–instrumental follow-up for previous HER2-positive BC pT2 pN1 (2017) treated with surgery, chemotherapy (epirubicin, cyclophosphamide and docetaxel), anti-HER2 therapy (trastuzumab) and hormone therapy (letrozole), due to an increase in CA15.3 to 58.90 UI/mL (normal value < 35) underwent 18F-FDG PET with findings of secondary bone localizations (spine and pelvis).

In her medical history, the patient had arterial hypertension and hypercholesterolemia treated with ramipril and atorvastatin, respectively. The patient is in excellent clinical condition (ECOG Performance Status 0) and is asymptomatic.

From June 2022 to October 2022, the patient underwent first-line chemotherapy with paclitaxel, pertuzumab and trastuzumab, and antiresorptive therapy with denosumab, with complete metabolic response on 18F-FDG PET and CA15.3 negativity; subsequently, the patient continued therapy with pertuzumab, trastuzumab, denosumab and fulvestrant. 

**Figure 1:**
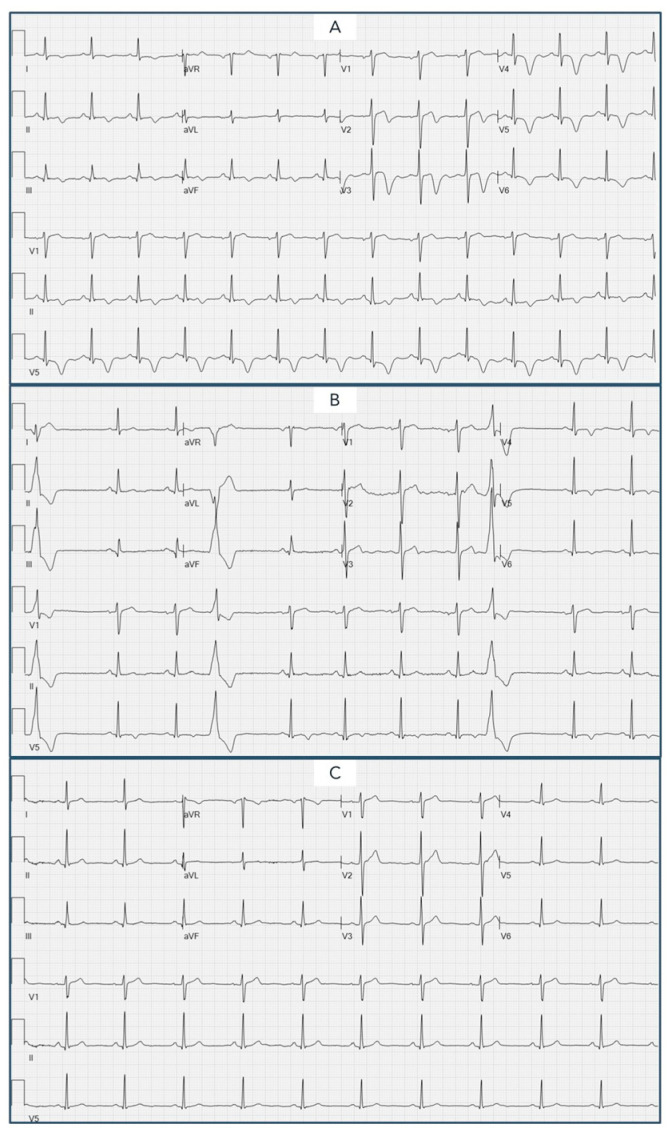
Evolution of 12-lead ECG. (**A**). ECG performed in the emergency department showing diffuse negative T wave and prolonged QTc interval. (**B**). ECG during hospitalization in the Coronary Unit evidencing an initial improvement in repolarization abnormalities and isolated monomorphic premature ventricular complexes. (**C**). ECG performed during follow-up showing the complete normalization of the previously described alterations.

On 31 March 2023, the patient entered the emergency department (ED) following the onset of typical chest pain with radiation to the left arm associated with autonomic symptoms, lasting approximately 50 min. The patient reported a similar episode approximately 2 days earlier. In the ED, she performed an ECG that highlighted the presence of diffuse negative T waves (Figure 1), and laboratory tests documented an increase in cardiac biomarkers.

Suspecting an acute coronary syndrome (ACS), the patient was admitted to our cardiac intensive care unit (CICU). 

**Figure 2:**
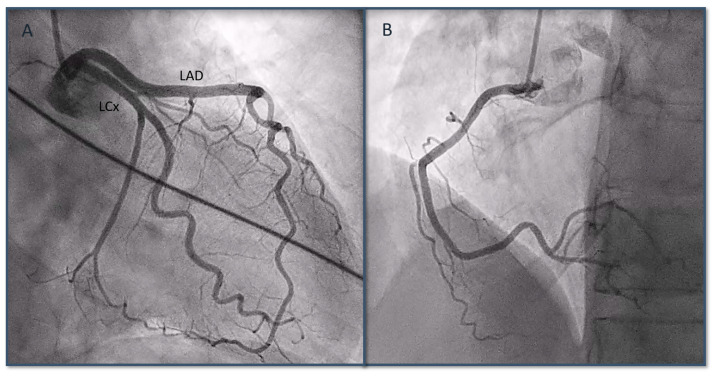
Coronary angiography showing absence of obstructive coronary artery disease in both left coronary artery (**A**) and right coronary artery (**B**). LAD: left descending anterior artery; LCx: left circumflex artery.

**Figure 3:**
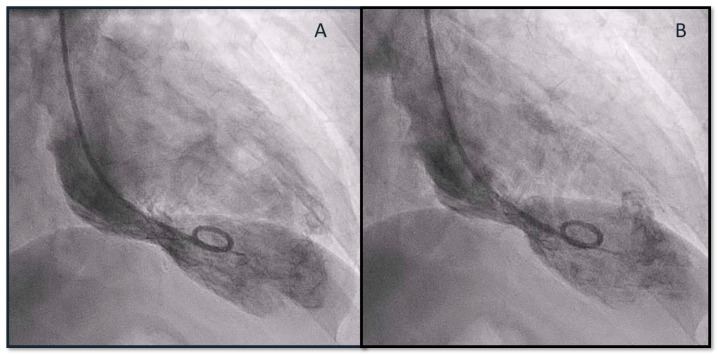
Cine-ventriculography at end-diastole (**A**) and end-systole (**B**) showing apical ballooning of apex.

A coronary angiography highlighted the absence of obstructive coronary lesions affecting the epicardial coronary vessels (Figure 2), and a simultaneous ventriculography evidenced hypokinesia of the mid-apical segments in toto and hyperkinesia of the basal segments, orienting the diagnosis toward TS (Figure 3).

During hospitalization, the patient maintained stable hemodynamic parameters and did not present further episodes of chest pain. The evolution of the ECG was characterized by QTc lengthening (486 ms), while TTE documented a slight reduction in LV ejection fraction (LVEF = 47%) in relation to hypokinesia of the apical segments of the LV. ECG monitoring did not document any significant arrhythmias, apart from isolated premature ventricular complexes (Figure 1).

**Figure 4:**
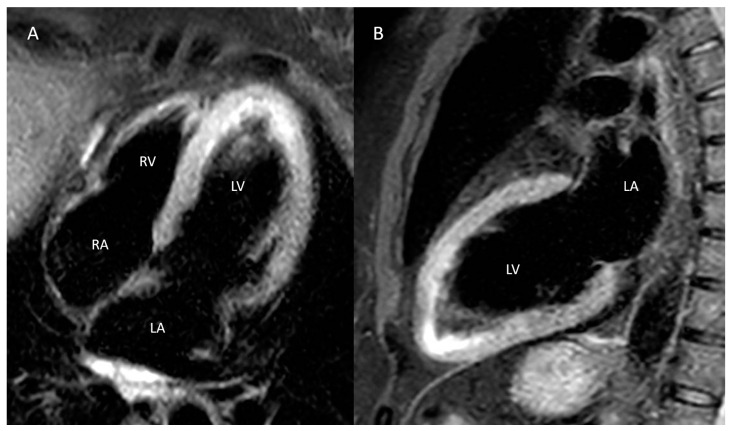
Cardiac magnetic resonance imaging: T2-weighted sequence showing the presence of diffuse edema of the apical segments in both the 4-chamber (**A**) and 2-chamber (**B**) long-axis views. LA: left atrium; LV: left ventricle; RA: right atrium; RV; right ventricle.

CMR with gadolinium performed during hospitalization confirmed the diagnosis of ST, highlighting the presence of diffuse edema in T2-weighted sequences in correspondence with the interventricular septum and the middle apical inferior wall, the apical anterior and lateral walls, and the apex, with concomitant alterations in regional contractility (Figure 4). On late post-contrast sequences, however, a focal subendocardial area in-keeping with a previous ischemic event was recognized in correspondence with the lower apical wall, with partial extension to the lower apical interventricular septum, whereas the absence of late gadolinium enhancement (LGE) with a non-ischemic pattern was documented.

The patient was discharged with an indication of single antiplatelet therapy with acetylsalicylic acid and anti-decompensation therapy with bisoprolol 2.5 mg/day. Furthermore, the patient continued her oncological therapy with only fulvestrant and denosumab. 

In July 2023, the patient underwent a control TTE, which highlighted the improvement in the LVEF (52%) with a persistence of hypokinesia of the lower interventricular septum and infero-lateral wall. On this occasion, the dosage of bisoprolol was uptitrated to 3.75 mg/day. 

Following the increase in CA15.3 to 60.50 U/mL, in July 2023, the patient resumed therapy with pertuzumab and trastuzumab, and continued fulvestrant and denosumab.

In August 2023, a further TTE was performed, which showed complete recovery of the biventricular systolic function (LVEF 55%). The ECG showed the complete normalization of the previously described alterations (Figure 1). 

No further episodes suggestive of cardiac toxicity have been recorded to date.

### 3.2. Systematic Review

TS caused by anti-HER2 therapy is rare [13]. 

The first case was reported in 1983 in Hiroshima City Hospital. Subsequently, it was diagnosed more frequently in Japan. Therefore, it was initially assumed that this pathology only affected people of Asian ethnicity, since TS was completely unknown to the Western world until the first case reports were published by French and American research groups in the late 1990s [14].

TS is characterized by acute and reversible symptoms (<21 days) related to LV dysfunction, with an initial clinical picture that mimics that of an ACS, i.e., sudden onset of [15] chest pain, dyspnea, palpitations, ECG abnormalities (ST segment elevation, Q waves and T waves with profound inversion and significant QT prolongation), increased troponin and akinesia of the mid-apical segments of the LV with “apical ballooning”, in the absence of significant atheromasia or signs of plaque instability on coronary angiography [7,13,16,17].

Unlike ACS, with the peak onset occurring in the early morning, TS occurs in the afternoon in most cases in conjunction with stressful events, i.e. emotional or physical, that act as triggers. 

The differential diagnosis of TS includes, in addition to ACS, esophageal spasm, gastroesophageal reflux, aortic dissection, myocarditis, acute pericarditis, pneumothorax, pulmonary embolism, Boerhaave syndrome (spontaneous esophageal rupture), cocaine-induced cardiomyopathy, dilated cardiomyopathy, hypertrophic cardiomyopathy and coronary artery spasm [9]. 

Although it is generally considered a benign condition given its reversible nature, in the acute phase the incidence of cardiogenic shock and death is like that in ACS. The high risk of hemodynamic and electrical instability, with the possible occurrence of potentially fatal arrhythmias, therefore requires intensive monitoring of vital parameters.

Due to apical akinesia, another possible complication is represented by the formation of thrombi at the apex of the LV in relation to blood stasis in correspondence to the walls affected by the regional dysfunctions. Thromboembolism is a complication that affects approximately 4% of patients with TS. 

Furthermore, basal hyperkinesis may result in dynamic left ventricular outflow tract obstruction (LVOTO), particularly in patients with basal septal hypertrophy, which further reduces stroke volume. Systolic anterior motion (SAM) of the anterior mitral valve leaflet may contribute to both LVOTO and the development of hemodynamically significant mitral regurgitation, present in 14–25% of patients with TS. 

Coronary angiography with left ventriculography is considered the diagnostic gold standard to exclude or confirm TS and differentiate it from ACS, as patients with TS do not present with any coronary lesions responsible for the kinetic alterations. 

Furthermore, ventriculography allows us to document the “apical ballooning” that occurs during systole. 

In the diagnostic work-up, CMR imaging with gadolinium is also recommended to exclude myocarditis and confirm the diagnosis of TS. In fact, this method, in addition to highlighting the wall motion abnormalities that most frequently involve the apex, allows the identification of areas of edema in T2-weighted sequences and the absence of fibrosis with a non-ischemic pattern in late post-contrast sequences [15].

The Mayo Clinic diagnostic criteria are the most widespread. 

Other research groups have proposed slightly different criteria for Takotsubo syndrome, namely the Japanese Guidelines, the Gothenburg criteria, the Johns Hopkins criteria, the proposal of the Takotsubo Italian Network, the criteria of the task force on TTS of the Heart Failure Association (HFA), the European Society of Cardiology (ESC) criteria and the criteria reported by Madias. Most recently, the InterTAK diagnostic criteria were published (Table 1). 

Based on the InterTAK criteria, depending on the presence or absence of some clinical, instrumental and laboratory elements, it is possible to establish the a priori probability of the syndrome and guide the subsequent diagnostic process (i.e., timing of coronary angiography; execution of coronary angiography vs. coronary CT) [14].

Although the typical apical form of TS is the most widespread variant, it is possible to observe forms with mid-ventricular involvement, basal forms or even forms characterized by focal alterations in segmental kinetics [18].

The pathogenesis of TS is unclear [7,9]. Some authors have classified TS into primary or secondary forms. Primary TS includes patients who develop the syndrome for an unclear reason, which may involve emotional stress. In secondary TS, a sudden activation of the sympathetic nervous system occurs, leading to an increase in circulating catecholamines, such as [15] in physical activities, medical conditions such as acute respiratory failure, pancreatitis, cholecystitis, pneumothorax, traumatic injury, sepsis, thyrotoxicosis, pregnancy, caesarean section, drowning, hypothermia, cocaine, alcohol or opioid withdrawal, carbon monoxide poisoning, nervous system conditions (for example stroke, head trauma, migraine, intracerebral hemorrhage or seizures), and also in malignant tumors (such as pheochromocytoma), including their treatments, such as chemotherapy and radiotherapy [9]. Stress, both psychological (primary form, up to ≈27% of cases) and physical (secondary form, up to ≈36% of cases), is responsible for the release of catecholamines, but in many cases it is not possible to identify a well-defined trigger [13,19]. Hypertension, hyperlipidemia, diabetes mellitus, smoking and a family history of cardiovascular disease are known risk factors for TS, but their implication in the pathogenesis of the syndrome is unclear [7,13,19]. While the literature agrees on the central role of sympathetic stimulation in the pathogenesis of TS, the mechanism by which catecholamines determine the regional wall motion abnormalities is unknown [7,20]. The most accredited hypothesis is that of direct toxicity of catecholamines on cardiomyocytes [2,21]. Along with the increase in the release of epinephrine and norepinephrine through the activation of the hypothalamic–pituitary–adrenal axis in response to a certain stress, other mechanisms of possible myocardial damage induced by circulating catecholamines include the induction of vasospasm of the epicardial vessels, direct myocardial stunning or acute and transient dysfunction of the microcirculation. 

After any stress and the release of catecholamines, the generalized impairment of endothelial function is a secondary effect of oxidative stress with high levels of endothelin or vasoconstrictor peptide, which can trigger vasospasm of multiple vessels including coronary and peripheral arteries. Catecholamine-mediated myocardial stunning can be partially explained by the β2-adrenergic receptor hypothesis. Higher densities of sympathetic nerve endings were observed in the basal myocardium compared to the apex. The position of the cardiac sympathetic nerve endings therefore does not explain the alterations in apical kinetics, which are more commonly found in TS; however, this mechanism may play a role in the pathogenesis of the basal variant. The acute peripheral circulatory and systemic responses after catecholamine release are accentuated with hypertensive peaks, which cause the myocardium to remain in a hyper-contractile state for a few minutes. When acute apical dysfunction develops on a background of normotension or hypotension, an unfavorable clinical evolution into cardiogenic shock with persistent vasoconstriction or paradoxical vasodilation is more frequent. The increase in intracavitary filling pressures leads to an increase in wall stress, which can contribute to the development of regional contractile dysfunctions. Furthermore, in TS, the endo-myocardial capillary density is substantially reduced, mainly due to the expansion of the extracellular matrix. Furthermore, the structure of cardiomyocytes alters, becoming enriched with intracellular vacuoles and ubiquitin, probably as a response to an oxygen deficit [22,23].

## 4. Discussion

TS is reported in cancer patients [24,25,26]. Cancer patients have a higher incidence of TS compared to the general population, with an average incidence of approximately 53 per 100,000 chemotherapy-related hospitalizations versus 20.4 in the general population, while its incidence in hospitalized patients with suspected acute coronary syndrome is approximately 2%. On the contrary, it has been observed that individuals with TS have a higher incidence of cancer than individuals without TS of the same age and sex, with an incidence ranging from 4% to 29%. The InterTAK registry reported that the malignancy most associated with TS is BC [27,28].

Furthermore, TS occurs more frequently in post-menopausal women (5.2/100,000 women versus 0.6/100,000 men) [22,23], like our patient. 

Estrogens play a cardioprotective role. In fact, cardiomyocytes express α and β estrogen receptors. The link between estrogen and the estrogen receptor increases the transcription of factors that play a cardioprotective role, such as heat shock protein and atrial natriuretic peptide, also protecting against the negative effects of catecholamines [7,9,14,15].

The occurrence of TS during oncological treatments is commonly attributed to the direct cardiotoxicity of the treatment (mainly through free radical-induced damage and the death of cardiac myocytes) [7,29].

Several chemotherapy regimens have been described in TS, such as 5-fluoracil, capecitabine, trastuzumab, bevacizumab, rituximab and more recently immunotherapy [15].

Carbone et al. reported that TS is associated with trastuzumab in 9.7% of published cases [30].

The only case of TS during pertuzumab and trastuzumab treatment, in association with nab-paclitaxel, was described by Lees et al. [11].

The peculiarity of our clinical case is that TS occurred during pertuzumab and trastuzumab treatment in association with an antiestrogen (fulvestrant), and not with a chemotherapy.

The mechanism underlying cardiotoxicity from anti-HER2 therapies likely involves HER2 signaling [31,32].

HER2 contributes to the survival of cardiomyocytes, and cardiomyocytes not stimulated by HER2 have a reduced response to excessive catecholamine release [33,34,35].

Other hypotheses have been described based on paraneoplastic phenomena or cancer-related stress (both psychological and physical, i.e. related to treatment or diagnostic procedures) [7,36].

TS in cancer patients has also been reported as a complication of specific tumors, such as pheochromocytoma and paraganglioma [27].

The prognosis is generally favorable, with mortality rates from 0% to 8% in systematic reviews, and in most cases, there is a complete recovery of the kinetic alterations within a few weeks; the relapse rate is around 5% within 3.8 years of diagnosis [7,10,22,37,38,39].

## 5. Conclusions

Although TS is usually a syndrome with reversible changes, its prognostic effect in cancer patients is questionable as it can lead to an interruption of anticancer therapy, which can negatively affect the oncological outcome. Therefore, rechallenge with the culprit antineoplastic therapy constitutes a serious clinical dilemma with limited safety data [27]. 

The average time to resume oncological treatment is approximately 20 days after the acute cardiac event, as the LVEF is generally recovered after this time interval [15].

If clinically essential, as in the present case, re-challenge with a responsible oncological treatment could be reconsidered after the complete normalization of the LV function with the patient under close clinical and instrumental cardiological follow-up [32].

## Figures and Tables

**Table 1:** InterTAK diagnostic criteria.

1. Transient left ventricular dysfunction (hypokinesia, akinesia or dyskinesia) presenting as either apical, midventricular, basal or focal wall motion abnormalities. The right ventricle may also be involved. Regional wall motion alterations generally extend beyond the supply territory of a single epicardial coronary vessel.
2. A physical, emotional or combined trigger may precede the acute event.
3. An acute cerebrovascular event (stroke, transient ischemic attack, subarachnoid hemorrhage, seizures) or the presence of pheochromocytoma may serve as a trigger.
4. Presence of new ECG changes (ST segment elevation or depression, inverted T waves, QTc prolongation).
5. Increase in cardiac biomarkers (troponin T or I, CK-MB, and NT-proBNP).
6. The presence of significant coronary artery disease does not exclude the diagnosis of TS.
7. Patients have no evidence of myocarditis.
8. Post-menopausal women are predominantly involved.

## Data Availability

Data are contained within the paper.

## Supplementary Materials

The following supporting information can be downloaded at https://www.mdpi.com/article/10.3390/biomedicines12010179/s1. Figure S1: Flow chart of the methodology.

## References

[1] Slamon D.J., Clark G.M., Wong S.G., Levin W.J., Ullrich A., McGuire W.L. (1987). Human breast cancer: Correlation of relapse and survival with amplification of the HER-2/neu oncogene. Science.

[2] Burgy M., Brossat H., Barthelemy P., Imperiale A., Trinh A., Hazam C.A., Bergerat J.P., Mathelin C. (2014). First report of trastuzumab treatment after postoperative Takotsubo cardiomyopathy. Anticancer Res..

[3] Baselga J., Perez E.A., Pienkowski T., Bell R. (2006). Adjuvant trastuzumab: A milestone in the treatment of HER-2-positive early breast cancer. Oncologist.

[4] Mantarro S., Rossi M., Bonifazi M., D’Amico R., Blandizzi C., La Vecchia C., Negri E., Moja L. (2016). Risk of severe cardiotoxicity following treatment with trastuzumab: A meta-analysis of randomized and cohort studies of 29,000 women with breast cancer. Intern. Emerg. Med..

[5] Swain S.M., Baselga J., Kim S.B., Ro J., Semiglazov V., Campone M., Ciruelos E., Ferrero J.M., Schneeweiss A., Heeson S. (2015). Pertuzumab, trastuzumab, and docetaxel in HER2-positive metastatic breast cancer. N. Engl. J. Med..

[6] Yu A.F., Manrique C., Pun S., Liu J.E., Mara E., Fleisher M., Patil S., Jones L.W., Steingart R.M., Hudis C.A. (2016). Cardiac Safety of Paclitaxel Plus Trastuzumab and Pertuzumab in Patients with HER2-Positive Metastatic Breast Cancer. Oncologist.

[7] Coen M., Rigamonti F., Roth A., Koessler T. (2017). Chemotherapy-induced Takotsubo cardiomyopathy, a case report and review of the literature. BMC Cancer.

[8] Tsuchihashi K., Ueshima K., Uchida T., Oh-mura N., Kimura K., Owa M., Yoshiyama M., Miyazaki S., Haze K., Ogawa H. (2001). Transient left ventricular apical ballooning without coronary artery stenosis: A novel heart syndrome mimicking acute myocardial infarction. Angina Pectoris-Myocardial Infarction Investigations in Japan. J. Am. Coll. Cardiol..

[9] Komamura K., Fukui M., Iwasaku T., Hirotani S., Masuyama T. (2014). Takotsubo cardiomyopathy: Pathophysiology, diagnosis and treatment. World J. Cardiol..

[10] Bybee K.A., Kara T., Prasad A., Lerman A., Barsness G.W., Wright R.S., Rihal C.S. (2004). Systematic review: Transient left ventricular apical ballooning: A syndrome that mimics ST-segment elevation myocardial infarction. Ann. Intern. Med..

[11] Lees C., Yazdan-Ashoori P., Jerzak K.J., Gandhi S. (2019). Takotsubo Cardiomyopathy During Anti-HER2 Therapy for Metastatic Breast Cancer. Oncologist.

[12] Sattar Y., Siew K.S.W., Connerney M., Ullah W., Alraies M.C. (2020). Management of Takotsubo Syndrome: A Comprehensive Review. Cureus.

[13] Matsumoto T., Oda T., Yoshida Y., Kimura S., Himei H., Tsuduki T., Takagi S., Takatani M., Morishita H. (2019). Takotsubo cardiomyopathy caused by infusion reaction to trastuzumab. Int. Cancer Conf. J..

[14] Ghadri J.R., Wittstein I.S., Prasad A., Sharkey S., Dote K., Akashi Y.J., Cammann V.L., Crea F., Galiuto L., Desmet W. (2018). International Expert Consensus Document on Takotsubo Syndrome (Part I): Clinical Characteristics, Diagnostic Criteria, and Pathophysiology. Eur. Heart J..

[15] da Silva Costa I.B.S., Figueiredo C.S., Fonseca S.M.R., Bittar C.S., de Carvalho Silva C.M.D., Rizk S.I., Filho R.K., Hajjar L.A. (2019). Takotsubo syndrome: An overview of pathophysiology, diagnosis and treatment with emphasis on cancer patients. Heart Fail. Rev..

[16] Yoshikawa T. (2015). Takotsubo cardiomyopathy, a new concept of cardiomyopathy: Clinical features and pathophysiology. Int. J. Cardiol..

[17] Kido K., Guglin M. (2017). Drug-Induced Takotsubo Cardiomyopathy. J. Cardiovasc. Pharmacol. Ther..

[18] Anderson R.D., Brooks M. (2016). Apical takotsubo syndrome in a patient with metastatic breast carcinoma on novel immunotherapy. Int. J. Cardiol..

[19] Sharkey S.W., Pink V.R., Lesser J.R., Garberich R.F., Maron M.S., Maron B.J. (2015). Clinical Profile of Patients with High-Risk Tako-Tsubo Cardiomyopathy. Am. J. Cardiol..

[20] Boland T.A., Lee V.H., Bleck T.P. (2015). Stress-induced cardiomyopathy. Crit. Care Med..

[21] Lyon A.R., Rees P.S.C., Prasad S., Poole-Wilson P.A., Harding S.E. (2008). Stress (Takotsubo) cardiomyopathy--a novel pathophysiological hypothesis to explain catecholamine-induced acute myocardial stunning. Nat. Clin. Pract. Cardiovasc. Med..

[22] Gopalakrishnan M., Hassan A., Villines D., Nasr S., Chandrasekaran M., Klein L.W. (2015). Predictors of short- and long-term outcomes of Takotsubo cardiomyopathy. Am. J. Cardiol..

[23] Ghadri J.R., Ruschitzka F., Lüscher T.F., Templin C. (2014). Takotsubo cardiomyopathy: Still much more to learn. Heart.

[24] Burgdorf C., Nef H.M., Haghi D., Kurowski V., Radke P.W. (2010). Tako-tsubo (stress-induced) cardiomyopathy and cancer. Ann. Intern. Med..

[25] Madias J.E. (2016). The intriguing triangle of cancer, chemotherapy and takotsubo syn-drome. Oxf. Med. Case Rep..

[26] Giza D.E., Lopez-Mattei J., Vejpongsa P., Munoz E., Iliescu G., Kitkungvan D., Hassan S.A., Kim P., Ewer M.S., Iliescu C. (2017). Stress-Induced Cardiomyopathy in Cancer Patients. Am. J. Cardiol..

[27] Keramida K., Farmakis D., Filippatos G. (2021). Cancer and Takotsubo syndrome: From rarity to clinical practice. ESC Heart Fail..

[28] Brunetti N.D., Tarantino N., Guastafierro F., De Gennaro L., Correale M., Stiermaier T., Möller C., Di Biase M., Eitel I., Santoro F. (2019). Malignancies and outcome in Takotsubo syndrome: A meta-analysis study on cancer and stress cardiomyopathy. Heart Fail. Rev..

[29] Smith S.A., Auseon A.J. (2013). Chemotherapy-induced takotsubo cardiomyopathy. Heart Fail. Clin..

[30] Carbone A., Bottino R., Russo V., D’Andrea A., Liccardo B., Maurea N., Quagliariel-lo V., Cimmino G., Golino P. (2021). Takotsubo Cardiomyopathy as Epiphenomenon of Car-diotoxicity in Patients with Cancer: A Meta-summary of Case Reports. J. Cardiovasc. Pharmacol..

[31] Hidalgo S., Albright C.A., Wells G.L. (2013). A case of trastuzumab-associated cardiomyopathy presenting as an acute coronary syndrome: Acute trastuzumab cardiotoxicity. Case Rep. Cardiol..

[32] Khanji M., Nolan S., Gwynne S., Pudney D., Ionescu A. (2013). Tako-Tsubo syndrome after trastuzumab—An unusual complication of chemotherapy for breast cancer. Clin. Oncol. (R. Coll. Radiol.).

[33] Lemmens K., Doggen K., De Keulenaer G.W. (2007). Role of neuregulin-1/ErbB signaling in cardiovascular physiology and disease: Implications for therapy of heart failure. Circulation.

[34] Gordon L.I., Burke M.A., Singh A.T.K., Prachand S., Lieberman E.D., Sun L., Naik T.J., Prasad S.V.N., Ardehali H. (2009). Blockade of the erbB2 receptor induces cardio-myocyte death through mitochondrial and reactive oxygen species-dependent path-ways. J. Biol. Chem..

[35] Volkova M., Russell R. (2011). Anthracycline cardiotoxicity: Prevalence, pathogene-sis and treatment. Curr. Cardiol. Rev..

[36] Madias J.E. (2015). Is Takotsubo syndrome in patients receiving chemotherapy drug-specific?. World J. Clin. Cases.

[37] Gianni M., Dentali F., Grandi A.M., Sumner G., Hiralal R., Lonn E. (2006). Apical ballooning syndrome or takotsubo cardiomyopathy: A systematic review. Eur. Heart J..

[38] Bybee K.A., Prasad A. (2008). Stress-related cardiomyopathy syndromes. Circulation.

[39] Golabchi A., Sarrafzadegan N. (2011). Takotsubo cardiomyopathy or broken heart syndrome: A review article. J. Res. Med. Sci..

