# Supplementary figures and images for: Takotsubo Syndrome during Pertuzumab and Trastuzumab Therapy for HER2-Positive Metastatic Breast Cancer
Source: Biomedicines. 2024 Jan 14;12(1):179. doi: 10.3390/biomedicines12010179 (PMC10813278; doi:10.3390/biomedicines12010179)

Figure S1. Flow chart of the methodology.

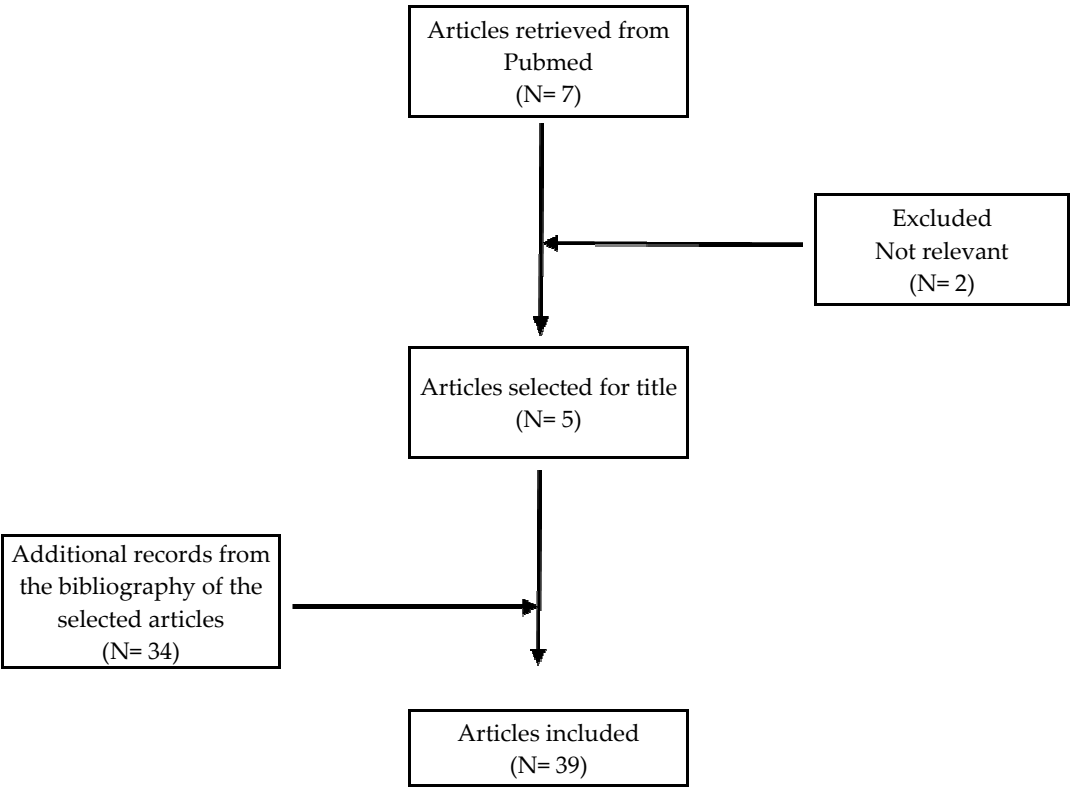

Supplement: Supplementary file 1 [file biomedicines-12-00179-s001.zip › biomedicines-2767357-SI.pdf]
